# Evolution of frustrated and stabilising contacts in reconstructed ancient proteins

**DOI:** 10.1007/s00249-021-01500-0

**Published:** 2021-02-11

**Authors:** Martina Crippa, Damiano Andreghetti, Riccardo Capelli, Guido Tiana

**Affiliations:** 1 Department of Physics and Center for Complexity and Biosystems, Università degli Studi di Milano and INFN, via Celoria 16, 20133 Milan, Italy; 2 Department of Applied Science and Technology, Politecnico di Torino, Corso Duca degli Abruzzi 24, 10129 Turin, Italy

**Keywords:** Protein evolution, Coevolutionary potential, Frustration, Reconstructed sequences

## Abstract

Energetic properties of a protein are a major determinant of its evolutionary fitness. Using a reconstruction algorithm, dating the reconstructed proteins and calculating the interaction network between their amino acids through a coevolutionary approach, we studied how the interactions that stabilise 890 proteins, belonging to five families, evolved for billions of years. In particular, we focused our attention on the network of most strongly attractive contacts and on that of poorly optimised, frustrated contacts. Our results support the idea that the cluster of most attractive interactions extends its size along evolutionary time, but from the data, we cannot conclude that protein stability or that the degree of frustration tends always to decrease.

**Supplementary Information:**

The online version contains supplementary material available at 10.1007/s00249-021-01500-0.

## Introduction

The evolutionary fitness of a protein is tightly related to its energetic properties. An important determinant of the evolutionary fitness of structured proteins is their thermodynamic stability. The stability requirement imposes a constraint to viable mutations and acts in a non-trivial, cooperative way at the level of the whole organism (Zeldovich et al. 2007; Rodrigues et al. 2016).

The stability of ancient proteins has been widely studied experimentally by sequence reconstruction, expressing and analysing ancient sequences obtained from extant protein families through maximum-likelihood or Bayesian methods (Wheeler et al. 2016). In general, ancient proteins are more stable than modern ones (Gaucher et al. 2008; Perez-Jimenez et al. 2011; Carstensen et al. 2012; Risso et al. 2013; Akanuma et al. 2013), although counterexamples exist (Hart et al. 2014). This behaviour is usually rationalised in terms of a higher environmental temperature in the Precambrian era (Boussau et al. 2008; Wheeler et al. 2016). Moreover, simple protein models suggest that the evolution of thermodynamic stability is controlled by a cluster of strongly stabilising residues that evolves in a slower way with respect to the others (Tiana et al. 2000, 2004a, b).

Another requirement that affects protein fitness is the kinetic accessibility of the native state, because a slow folding rate would increase the risk of misfolding and aggregation. Since proteins are frustrated systems, namely unfavourable interactions linger in their energy ground state (Ferreiro et al. 2014), they would be expected to display slow, non-exponential kinetics (Bryngelson and Wolynes 1989). It was suggested that evolution minimises the degree of frustration of proteins to avoid kinetic traps (Bryngelson and Wolynes 1987). Even in the absence of consistent frustration, the folding process is regulated by an entropic barrier that determines folding rate. Such a barrier is usually overcome by nucleation of specific parts of the protein chain (Wetlaufer 1973; Abkevich et al. 1994). Both the folding nucleus (Mirny and Shakhnovich 2001) and the folding rate (Tzul et al. 2017) are usually highly conserved along evolutionary time.

The goal of the present work is to study the network of interactions between amino acids in the native state of reconstructed ancient proteins. In particular, we focused on the evolution of the network of strongly attractive two-body contacts, which stabilises their native state, and on frustrated contacts, which affect folding rates; the main intent is to study how contacts evolve as a function of evolutionary time. Understanding the evolution of the elements which stabilise proteins can be relevant both for fundamental reasons, for designing new proteins and for predicting the microbial resistance to drugs and vaccines (Russ et al. 2020).

A key problem in pursuing this goal is the quantification of the interaction energies between amino acids. The majority of classical force fields are atom-based and usually require an explicit description of the dynamics of the solvent, thus are not easy to use for our purposes. We then chose to describe the interaction between amino acids with a 2-bodies potential whose parameters are obtained from correlations between mutations in alignments of extant homologous sequences (Morcos et al. 2011). In brief, the sequences of homologous sequences are regarded as equilibrium realisations of a Potts model of unknown parameters. Using techniques of inverse statistics, one can look for the best choice of the interaction energies that are compatible with the empirical correlation functions (Nguyen et al. 2017). Different approximations can be employed to implement the inversion (Morcos et al. 2011, 2013; Ekeberg et al. 2013; Figliuzzi et al. 2015; Cuturello et al. 2020), which anyway perform similarly to each other in obtaining the interaction energies (Franco et al. 2019). For this reason, we employed the original mean-field procedure (Morcos et al. 2011) because of its computational efficiency. This strategy of calculation of the interactions proved efficient in predicting the native conformation protein monomers (Morcos et al. 2011) and dimers (dos Santos et al. 2015), of their conformational fluctuations (Jana et al. 2014; Sutto et al. 2015), to study protein aggregation (Tian et al. 2015; Kassem et al. 2018), the effect of mutations in protein stability (Lui and Tiana 2013; Contini and Tiana 2015), the identification of protein domains (Halabi et al. 2009; Granata et al. 2017) and the identification of interaction hotspots in transmembrane proteins (Baldessari et al. 2020). The use of coevolutionary data to simulate protein evolution (de la Paz et al. 2020) was also useful to investigate the details of neutral evolution theory.

In this work, we analysed five protein families, reconstructing their evolution and calculating the interaction network in every extant and reconstructed molecule, for a total of 890 proteins. We then analysed how the interaction network depends on evolutionary time.

## Calculation of interaction energies along evolution

We studied the evolution of the energetic properties of *β*-lactamase (BLM), thioredoxin (TRD), nucleoside-diphosphate kinase (NDK), cytochrome c (CYC) and ribonuclease H (RDH). These are well-characterised protein families, they are evolutionarily quite old and contain a large number of sequences.

From the alignment of the extant proteins in each family, we calculated the interaction tensor *ε*_*ij*_(*σ*, *ρ*) with the mean-field approach of ref. (Morcos et al. 2011), which is remarkably fast. The key idea is that pairs of residues which are close in space and that attract strongly each other undergo highly correlated mutations (while each of the two residues are not necessarily more conserved, see below). Inverting the problem, most correlated residues are expected to be close in space and strongly interacting, and this interaction can be quantified within an inverse Potts model (Nguyen et al. 2017).

Operatively, the “full” alignments are obtained from the Pfam database (Punta et al. 2012), those with less than 30% gaps are retained and those with sequence identity larger than 70% were down-weighted as in ref. (Morcos et al. 2011). For all families, there are more than 10^4^ sequences (cf. Table 1). We calculated one- and two-point frequencies using pseudocounts on the overall fraction of residues types (*x* = 0.5), on the fraction of residues types in the alignment (*y* = 0.1) and on the fraction of residues type in the specific position (*z* = 1.0) as in ref. (Lui and Tiana 2013).

**Table 1:** List of protein families used in this study

Protein family	PFAM code	# Pfam seq	# extant seq	# reconstr. seq	AA length
*β*-Lactamase (BLM)	PF00144	36325	31	40	267
Thioredoxin (TRD)	PF00085	59245	92	95	107
Nucleoside-diphosphate kinase (NDK)	PF00334	11973	143	162	135
Cytochrome c (CYC)	PF00034	23855	66	81	104
Ribonuclease H (RNH)	PF00075	14837	75	105	151

Energies are expressed in units of the evolutive temperature (Shakhnovich and Gutin 1993a), which cannot be determined within the model and which does not relate straightforwardly to the environmental temperature, being expected to be smaller than that (Morcos et al. 2014). They are gauged setting to zero the interaction of the gap, regarded as the 21st type of residue, with all the others (Lui and Tiana 2013). The (*N* × *N* × 21 × 21)-tensor contains the contact energies between all pair of sites for any kind of amino acid that they can host.

The sequence of ancient proteins of each family is reconstructed through a maximum-likelihood scheme with PAML (Yang 2007) from the Pfam alignment and from the phylogenetic tree that defines the links between proteins over time (see, e.g. Fig. S1 in the Supp. Mat.). We selected a subset of extant sequences from the Pfam alignment that have mutually less than 20% of gaps (cf. Table 1), selecting a single protein per organism. When multiple proteins are associated with the same organism, that with minimum number of gaps is preferred. We then built a tree that defines the relationship between the selected organisms which host the proteins from TimeTree (Hedges et al. 2015), that also gives an estimate of the age of each reconstructed ancient sequence. The sequences of the proteins corresponding to the nodes of the tree, that is the ancestors of extant proteins belonging to the Pfam dataset, are then reconstructed with PAML. The resulting alignment is free of gaps.

For any sequence of a family, putative native conformations are predicted by homology modelling using Modeller (Webb and Sali 2016). Homologs of known structure are selected with *E *value < 0.01, giving a variable number of templates, usually between 1 and 5. Subsequently, the obtained structure is optimised through a short minimization run with Gromacs (Van Der Spoel et al. 2005) using the Amber99SB force field.

The energy tensor is then filtered, setting to zero the elements that are not in contact in the (crystallographic or putative) native structure. Two residues are assumed to be in contact if their *C*_*β*_ (*C*_*α*_ in case of glycine) are closer than 6.5 Å.

As a consequence of this procedure, the energy tensor *ε*_*ij*_(*σ*, *ρ*) is the same for all proteins of each family (but in general different between different families); on the other hand, the projection of the four-dimensional tensor on the specific sequence to obtain the two-dimensional interaction matrix *ε*_*ij*_ between its amino acids depends on the specific protein.

**Fig. 1:**
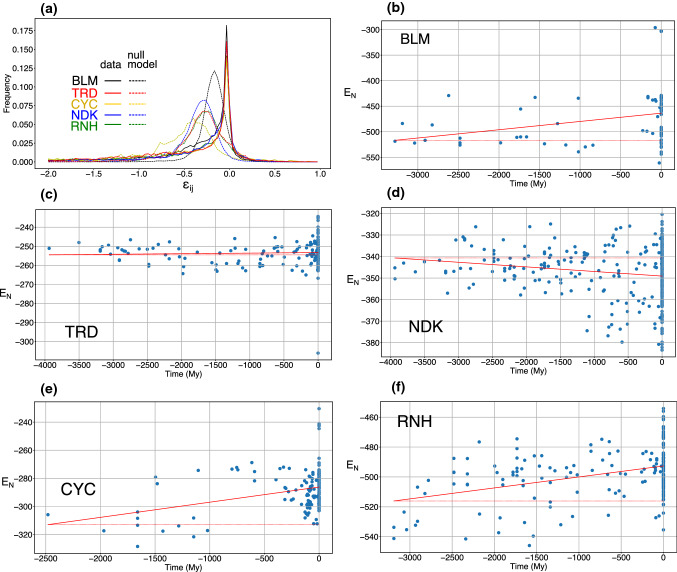
**a** With solid lines, the distribution of the interaction energies *ε*_*ij*_ for the native contact of all family members of *β*-lactamase (BLM), thioredoxin (TRD), nucleoside diosolphate kinase (NDK), Cytochrome c (CYC) and Ribonuclease H (RDH). Dashed lines indicate the energies obtained from a random bootstrap of the sequences. **b**–**f** the native energy *E*_*N*_ of the extant and reconstructed proteins of each family as a function of evolutionary time; the continuous line is the linear fit, while the dashed line is a horizontal reference

The distribution of the native contact energies between residues in all proteins belonging to each family is displayed in the upper-left panel of Fig. 1. It displays a sharp peak centred in zero and a long tail towards negative values. The distribution of energies over all sequences of a family is similar to that of single sequences (cf. Fig. S2 in the Supp. Mat.), so it can be regarded as representative of any sequence.

In Fig. 1a, it is also displayed the distribution of energies associated with a null model, obtained from a bootstrap procedure in which the residues at each position are randomly reshuffled among the sequences (thus keeping one-site frequencies unchanged).

While the distribution of energies in the null model is rather symmetric, Gaussian-like and centred around negative values, that of protein energies displays a long tail towards negative elements, stemming from a sharp peak centred close to zero. Energies in proteins seem, thus, much more polarised than in the null model. On the side of positive energies, the distributions associated with the five protein families do not display any tail but a decay similar to that of the null model.

Considering that the distribution displayed in Fig. 1a is limited to interactions that are in contact in the native conformation, its shape supports the idea that proteins are stabilised by a core of strong interactions (those belonging to the negative tail of the distribution), that constrain the rest of weakly interacting residues, corresponding to the peak around zero (Tiana et al. 1998; Mirny and Shakhnovich 1999). This shape is also consistent with the asymmetrical distribution of mutational energies obtained for several proteins (Tokuriki et al. 2007).

## Thermodynamic stability of ancestral proteins

The stability of a protein is essentially determined by the energy of its native state, because the competing, denatured states are self-averaging, i.e. their thermodynamic properties do not depend on the detailed sequence (Shakhnovich and Gutin 1993a, b). In Fig. 1, we plotted the native energies *E*_*N*_ of extant and reconstructed proteins as a function of evolutionary time. This quantity is calculated simply summing all the coevolutionary energies of pairs of residues that are in contact; the lower the value of *E*_*N*_, the more stable is the protein.

It should be noted that the self-averaging character of the denatured state can be guaranteed only if the composition of the protein in terms of type of amino acids, especially in terms of hydrophobic residues, remains constant. The reconstructed proteins display a rather constant composition (cf. Fig. S3 in the Supp. Mat.) and, thus, comply with this requirement.

The trend of *E*_*N*_ appears as system dependent. In the case of BLM, CYC and RNH, stability decreases towards recent proteins. In this case, the slope obtained from a linear fit of the energies as a function of time is significantly larger than that of a null model obtained from a random bootstrap of the calculated energies; also Kendall’s tau test indicates significant monotonicity (cf. the *p* values in Table 2). Also a different null model in which we calculate the energies of a random set of sequences gives in all cases a constant temporal trend, with standard deviation on the slope < 10^–5^ My^−1^ (cf., e.g. Fig. S4 in the Supp. Mat.).

**Table 2:** Summary of the energetic features of the protein families

Family	Std. dev.	Slope	*p* value (bootstrap)	*p* value (Kendall)
BLM	55.8	0.02	**0.038**	**0.030**
TRD	6.6	3.0 × 10^−4^	0.259	0.084
NDK	12.8	− 2.1 × 10^−3^	**0.001**	0.053
CYC	14.6	0.011	**< 10**^**–6**^	**0.003**
RNH	17.5	8.3 × 10^−3^	**< 10**^**–6**^	**7.8** × 10^−5^

It is listed the standard deviation of the native energies *E*_*N*_, the slope of the linear fit (cf. solid line in Fig. 1), the *p* value associated with the slope and calculated with a bootstrap and the *p* value associated with Kendall’s tau (the most significant are in bold)

In the case of NDK, the decreasing values of *E*_*N*_ indicate a significant increase of stability with time; while for TRD, we cannot spot any monotonic behaviour. Anyway, for all the considered proteins, the variability of *E*_*N*_ is quite large (cf. the standard deviation in Table 2), even at similar times. The variability within different kingdoms is the same as that in the overall set of proteins (cf. Fig. S5 in the Supp. Mat.).

**Fig. 2:**
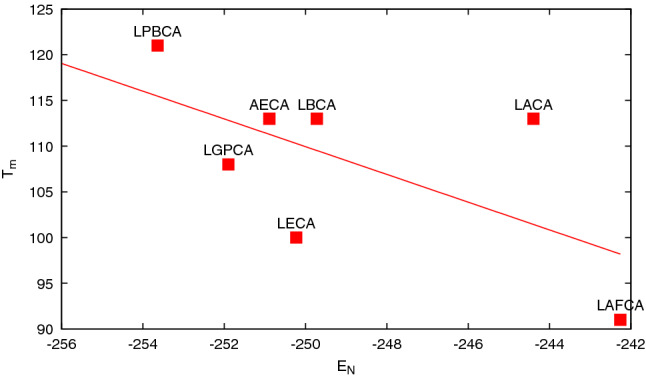
The denaturation temperatures *T*_*m*_ of the seven reconstructed TRD proteins of ref. (Perez-Jimenez et al. 2011) as a function of the native energy *E*_*N*_ calculated with the present model. The labelling corresponds to that of the referenced article. The dashed line indicates a linear fit, expected for a two-state model. The correlation coefficient is − 0.63. The increase of energy with respect to time has a slope of 0.6 My^−1^

The overall tendency of proteins to destabilise towards the present age (but with several exceptions) has been already recognised (Wheeler et al. 2016). This tendency was explained either as a selective advantage of marginally stable proteins in terms of adapting to new functions (Bloom et al. 2004) or as an entropic effect in sequence space (Taverna and Goldstein 2002). However, at variance with our findings, the reconstructed proteins belonging to the TRD and NDK families display decreasing stability as measured by differential scanning calorimetry (Perez-Jimenez et al. 2011) and circular dichroism (Akanuma et al. 2013), respectively. One should consider that these conclusions are drawn by the analysis of 7 and 12 proteins, respectively, which is a small subset of the 187 and 305, respectively, considered in our analysis. For example, the analysis of the of the TRD proteins reconstructed in ref. (Perez-Jimenez et al. 2011) shows that the experimental denaturation temperature is negatively correlated with the predicted native-state energy (cf. Fig. 2), as expected for the equilibrium of a two-state system, and thus, the model predicts for these seven proteins a decreasing stability along evolutionary time. This means not only that the model is able to predict the thermodynamic properties of proteins characterised by calorimetry, but also that selecting few proteins one can observe a trend that is different from that of the whole set. Note that filtering only the energies associated with native contacts is useful, because not doing it leads to an unphysical positive correlation between denaturation temperature and native energy (cf. Fig. S6).

## Evolution of strongly attractive contacts

We defined operatively “strongly attractive” contacts as those with energy *ε*_*ij*_ below a threshold *ε*_th_ such that globally 5% of the contacts of the null models of the five proteins lie below *ε*_th_. Using the energies displayed in Fig. 1, we found that *ε*_th_ = − 0.57.

**Fig. 3:**
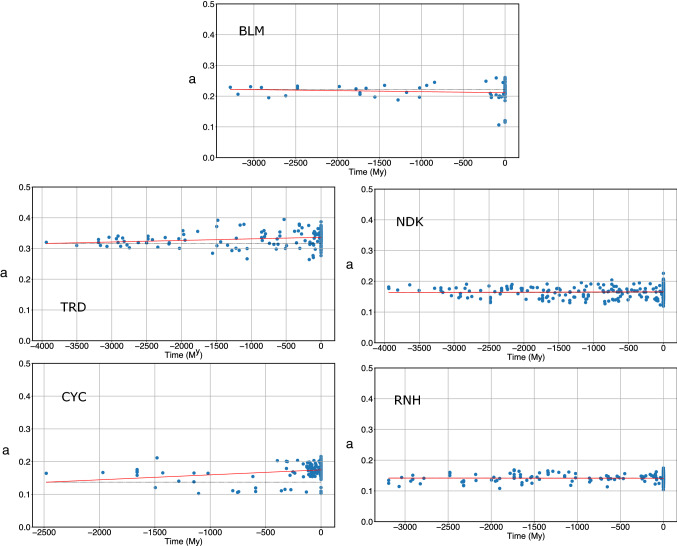
The fraction a of strongly attractive contacts, defined as those contacts whose energy is lower than *ε*_th_, for extant and reconstructed proteins

The fraction *a* of strongly attractive contacts of extant and reconstructed proteins is displayed in Fig. 3. The value of *a* is significantly increasing for TRD and CYC (cf. Table 3). A linear fit of *a* as a function of time gives for these two proteins an increase rate of the order of 10^–5^ year^−1^. The *p* values obtained calculating the slopes of randomly bootstrapped data are 0.002 and 0.001, respectively. We also computed the *p* values associated with the null hypothesis that the increase in *a* is not monotonic, using Kendall’s tau. The monotonicity of *a* is significant as well (cf. Table 3).

**Table 3:** Summary of the evolution of strongly attractive contacts for the five protein families (first column)

Family	*a*			LCS		
	Slope	*p* (bootstrap)	*p* (Kendall)	Slope	*p* (bootstrap)	*p* (Kendall)
BLM	− 3.3 × 10^−6^	0.16	0.23	4.2 × 10^−5^	**0.006**	**0.002**
TRD	5.1 × 10^−6^	**0.002**	**0.006**	5.8 × 10^−5^	**< 10**^**−6**^	**2 × 10**^**−8**^
NDK	6.4 × 10^−7^	0.27	0.04	1.4 × 10^−5^	**0.05**	0.2
CYC	1.5 × 10^−5^	**0.001**	**0.003**	3.9 × 10^−5^	**0.007**	**0.004**
RNH	− 1.6 × 10^−7^	0.45	0.11	2.7 × 10^−5^	**0.0001**	**3 × 10**^**−5**^

It is reported for the fraction *a* of strongly attractive contacts and for the largest-cluster size (LCS) the slope of the linear fit and the *p* values associated with a bootstrap of the protein age and with Kendall’s tau test

The most significant are in bold

For the other three protein families (BLM, NDK and RNH), no statistically significant trend could be identified.

## Interaction network analysis

**Fig. 4:**
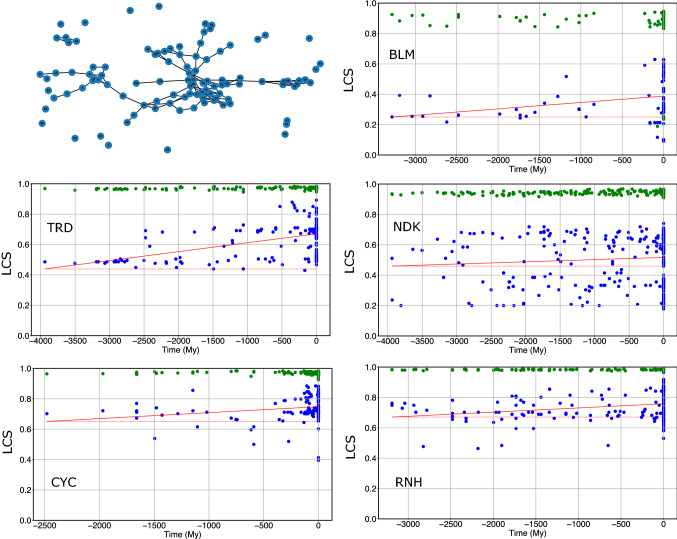
The upper-left panel is an example of network of strongly attractive interactions of BLM. The other panels display (in blue) the largest-cluster size (LCS), normalised to the total number of nodes, of extant and reconstructed proteins. Green points indicate the mean LCS of proteins with randomly reshuffled strongly attractive contacts

We then analysed the network whose nodes are all the amino acids of each protein and whose links are the strongly attractive contacts (see, e.g. upper-left panel of Fig. 4). All networks display one, or few, large clusters and several orphans (i.e., nodes without links). The largest-cluster size (LCS) is in all cases significantly smaller than that of randomly generated proteins (*p* value < 10^–6^ and green points in Fig. 4). LCS increases from more ancient to more recent proteins (see blue points in Fig. 4) for all families in a statistically significant way, as calculated from a random bootstrap of the energies of reconstructed proteins (see Table 3). Also, the comparison with randomly generated sequences give *p* values < 10^–6^ (see Fig. S7 in the Supp. Mat.). The number of orphans and the clustering coefficient are significantly larger than those expected from random networks, but they do not display a regular temporal trend (cf. Fig. S8 in the Supplementary Material). These data agree with the literature that all studied proteins display a core of contacts (Mirny and Shakhnovich 1999; Tokuriki et al. 2007) that strongly stabilise the native state (often, but not always hydrophobic) and suggest that, although the total number of strongly attractive contacts does not always increase along evolution, the set of strongly stabilising residues does.

The above results are quite robust with respect to the threshold ε_th_ used to define the strongly attractive contacts (cf., e.g. Fig. S9 in the Supp. Mat.).

**Fig. 5:**
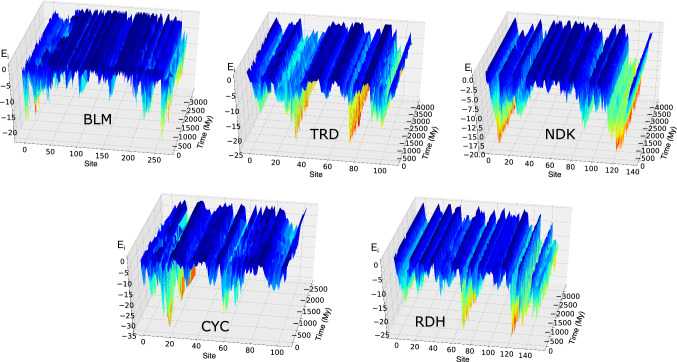
The evolution of strongly interacting contacts in each site of the five families under study

The evolution of the position of the amino acids involved in the strongly attractive contacts can be found in Fig. 5, that displays the sum *E*_*i*_ of attractive interactions for each site *i*. One can notice that strongly interacting sites (whatever are the residues hosted there) are rather conserved. Those present in ancient proteins tend to remain in extant proteins and sometimes new ones are added during evolution.

Interestingly, the correlation between strongly interacting and highly conserved residues in the alignment is poor (cf. Fig. S10 in the Supp. Mat.). In particular, there are many more highly conserved sites than strongly interacting sites, suggesting that there could be several reasons why residues are evolutionary conserved (Mirny and Shakhnovich 1999).

## Evolution of frustrated contacts

Frustration is the property of some complex systems not to be able to get rid of unfavourable interactions even in the ground state. As discussed by Phil Anderson in ref. (Anderson 1978), it is not straightforward to quantify if a system is frustrated if not for spin systems. His suggestion was to identify the ground state of the system, to partition it into subsystems and to quantify the scaling of the interaction energy between them as a function of the area of the separation surface. For finite-range interactions, as those acting between amino acids, the scaling is expected to be linear if the system is ferromagnetic-like. If it is frustrated, one expects a sub-linear scaling because of the compensation between attractive and repulsive interactions.

**Fig. 6:**
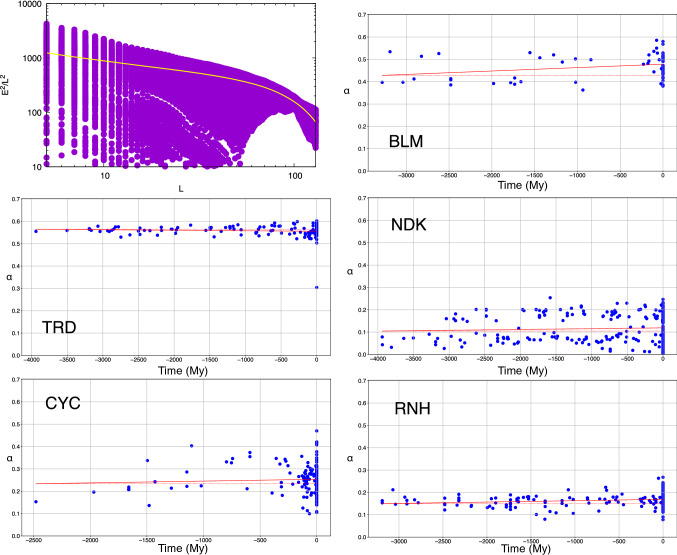
The scaling of the square interfacial energies *E*^2^ between segments of a BLM (GI number: gi116251120) of length *L* and the rest of the protein as a function of *L*, normalised by *L*^2^ and plotted in logarithmic scale. The dashed line is the mean square energy. The other plots display the scaling coefficient *α* of the mean square energy, calculated in the linear region, for the proteins of the five families

In the upper-left panel of Fig. 6 it is displayed, as an example, the square of the interfacial energy between the segments of various length *L* of a BLM and the rest of the protein. The fact that the mean square energy $\overline{{E^{2} }} (L)$ is a decreasing function supports the accepted idea that proteins are frustrated systems.

The shape of $\overline{{E^{2} }} (L)$ displays a power law ${1/L}^{\alpha }$ followed by a drop. The largest is $\alpha$, the more frustrated is the system; $\alpha =0$ for a ferromagnetic-like system. The values of $\alpha$ for the extant and reconstructed proteins are displayed in the various panels of Fig. 6. In the case of BLM and RNH, the degree of frustration increases with time in a significant way; while for the other three analysed proteins, a specific trend cannot be established (cf. the *p* values in Table 4).

**Table 4:** The slope of the linear regression of the number of the scaling coefficient *α* of the interfacial energies and of the number of frustrated contacts as a function of evolutionary time and the *p* values calculated on the slopes with a random bootstrap and with Kendall’s tau test

Family	*α*			# frustrated contacts		
	Slope	*p* (bootstrap)	*p* value (Kendall)	Slope	*p* (bootstrap)	*p* value (Kendall)
BLM	1.5 × 10^−5^	**0.009**	0.23	3.5 × 10^−7^	0.06	0.41
TRD	− 2.2 × 10^−6^	0.095	0.43	− 6.2 × 10^−6^	0.012	**< 10**^**–6**^
NDK	3.6 × 10^−6^	0.17	0.36	6.4 × 10^−7^	0.09	0.29
CYC	8.0 × 10^−6^	0.25	0.45	1.5 × 10^−5^	0.006	**0.001**
RNH	6.1 × 10^−6^	**0.006**	**0.009**	− 1.6 × 10^−7^	0.22	0.4

**Fig. 7:**
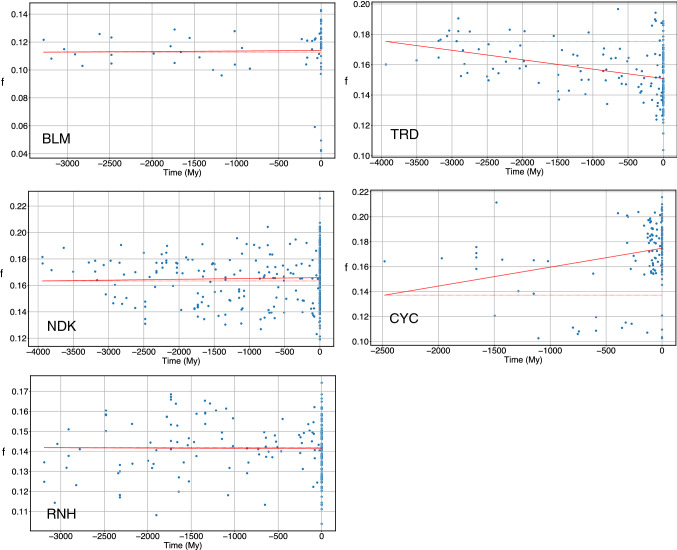
The number *f* of frustrated contacts as a function of evolutionary time. The solid line indicates the result of a linear fit

Another way of quantifying the degree of frustration is counting the number of contacts with energy *ε*_*ij*_ > 0, thus relying on the gauge we chose that sets the zero to the interaction of any residue with gaps. The fraction *f* of frustrated contacts over the total number of contacts is displayed in Fig. 7. TRD and CYC have a significant monotonic behaviour, decreasing for the former and increasing for the latter; while the other proteins do not show significant monotonicity (cf. the *p* values in Table 5; the most significant are in bold).

**Table 5:** The slope and the *p* values associated with the number of orphans and the clustering coefficient

Family	# of orphans			Clustering coefficient		
	Slope	*p* (bootstrap)	*p* value (Kendall)	Slope	*p* (bootstrap)	*p* value (Kendall)
BLM	5.8 × 10^−6^	0.26	0.10	0.002	0.14	0.4
TRD	8.7 × 10^−7^	0.37	0.76	− 5.8 × 10^−6^	**0.04**	**0.02**
NDK	− 8.2 × 10^−6^	**0.001**	**7.1** × 10^−5^	− 4.5 × 10^−6^	**0.01**	**0.08**
CYC	− 3.1 × 10^−5^	**0.017**	**0.003**	− 3.9 × 10^−6^	0.21	0.24
RNH	2.0 × 10^−6^	0.29	0.09	3.2 × 10^−7^	0.47	0.25

The most significant are in bold

Notice that $\alpha$ and *f* do not provide exactly the same information, because the scaling of the interfacial energy depends not only on the number of frustrated contacts but also on their spatial arrangement.

Overall, these data do not support a regular evolutionary trend for the number of frustrated contacts.

**Fig. 8:**
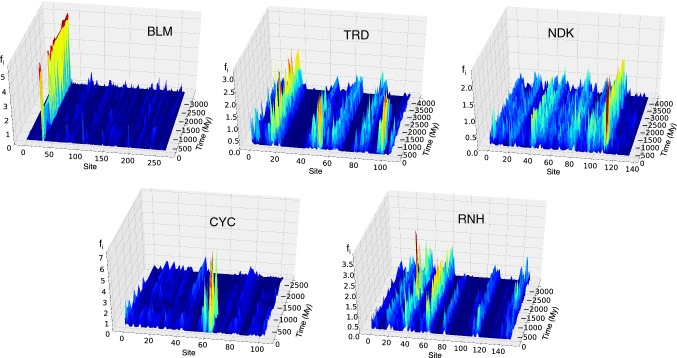
The number *f*_*i*_ of frustrated contacts in each site i of the protein as a function of evolutionary time, averaged over all proteins in the same age

We then studied how frustration is localised within proteins. In Fig. 8, it is shown for each family the number *f*_*i*_ of frustrated contacts in each site, averaged over all proteins of the same age. All proteins appear to display few sites concentrating most frustration and these sites are highly conserved along evolution. In few cases (three in TRD, three in CYC, one in RNH) sites that were not frustrated in more ancient proteins become frustrated. The opposite is never observed.

**Fig. 9:**
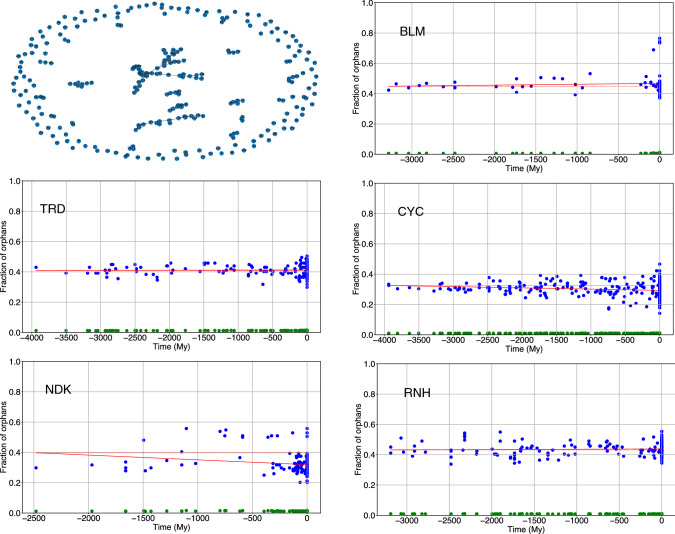
In the upper-left panel, the network of frustrated interactions of TRD. The other panels show the fraction of orphans as a function of evolutionary time (blue points). Green points are a negative control obtained from a random reshuffling of contacts

Frustrated contacts tend to aggregate into clusters, see the network of frustrated interactions in Fig. 9. As a result, most amino acids are not connected by frustrated contacts, i.e. they are “orphans”. The number of orphans is much larger than that one would observe in a random graph with the same number of nodes and links (*p* value < 10^–6^, see also Fig. 9). In fact, random networks display a wide distribution of cluster sizes with a negligible number of orphans (see, e.g. Fig. S11 in the Supp. Mat.), while proteins display few main clusters and a large number of orphans. The amount of orphans either decreases with time (as in CYC and NDK) or fluctuates non-monotonically (as BLM, TRD and RNH, see Fig. 9 and the *p* values in Table 5).

The size of the largest cluster is comparable to the one of a random network and does not display a regular trend with respect to evolutionary time (cf. Fig. S12 in the Supp. Mat.). On the other hand, its clustering coefficient is significantly larger than that of a random cluster and either decreases significantly with time (for TRD and NDK) or does not display a specific trend (for the other proteins, cf. Fig. S13).

Summing up, frustrated contacts concentrate into few clusters that are not particularly large but are highly connected. There is a signal, although weak, that this tendency increases towards more recent proteins.

A popular tool to study frustration contacts within proteins is the frustratometer developed by Ferreiro and coworkers (Parra et al. 2016). We have performed a rough comparison of the pattern of frustrated contacts of the coevolutionary model with that obtained from the online version of the frustratometer. The results of the comparison are highly family dependent; for example, they are quite good in the case of TRD and much worse for NDK (cf. Fig. S14 in the Supp. Mat.).

## Discussion and conclusions

We studied the energetic properties of five protein families and of their reconstructed ancestors; that is in total 890 proteins. The two key tools that permitted this analysis are coevolutionary potentials and the reconstruction algorithms of ancient proteins. Coevolutionary potentials are an efficient and realistic way of describing the interactions that stabilise proteins at the scale of amino acids. They are predictive of many protein features (Halabi et al. 2009; Morcos et al. 2011, 2013; Lui and Tiana 2013; Jana et al. 2014; Tian et al. 2015; Contini and Tiana 2015; dos Santos et al. 2015; Sutto et al. 2015; Granata et al. 2017; Kassem et al. 2018; Baldessari et al. 2020), but need large sequence alignments as input, and, thus, cannot be applied to all protein families. Moreover, they suffer of systematic errors in estimating the interaction energy of residues involved in active sites, that could coevolve for reasons which are not related to the stabilisation of the native state and, thus, cannot be described in terms of the inverse Potts model. In fact, while one would expect that the interactions of the active site can be frustrated, the model predicts erroneously strongly attractive interactions (see Fig. S15 in the Supp. Mat.).

Also, the maximum-likelihood reconstruction of ancient proteins is powerful but not error free. In fact, it was pointed out that when different stabilising mutations accumulate along different lineages, the maximum-likelihood reconstruction could incorrectly incorporate all of the stabilising mutations in the same ancient sequence, resulting in an over-stabilised ancestor (Wheeler et al. 2016). An indication that this is not the case here is that in ancient reconstructed sequences, we do not observe an increase of hydrophobic residues, which are expected to be the most stabilising ones. Anyway, we observe various types of behaviour, with families becoming more stable and families becoming less stable along evolution. In the literature, the large majority of proteins reconstructed by maximum-likelihood algorithms and studied biochemically were shown to become less stable towards recent times (Gaucher et al. 2008; Perez-Jimenez et al. 2011; Carstensen et al. 2012; Risso et al. 2013; Akanuma et al. 2013); this behaviour was explained by an increased environmental temperature in the pre-Cambrian era, which imposed a larger stability to proteins. However, these studies involved few proteins, to be compared with our hundreds. Moreover, we have also shown that the variability in thermodynamic stability is very large even in proteins of similar ages, and consequently drawing general conclusions from a small sampling is quite dangerous.

We then focused the attention to the contacts which mostly stabilise the native state of the proteins. Their number does not seem to vary systematically along evolution. However, they form in each protein a small, highly interconnected cluster. The size of this cluster increases towards recent times, including more and more residues. This is in agreement with the observation that more recent proteins display a more highly connected core (Tiana et al. 2004b). The analysis of amino-acid mutations (Tokuriki et al. 2007) and of protein models (Tiana et al. 2004c) suggests that the stabilisation energy in proteins is not distributed uniformly but is concentrated in a small core. This analysis of reconstructed proteins suggests that the size of the core increases with time.

Also frustrated interactions, that is interactions that evolution could not optimise, are an important feature of proteins. We quantified the degree of frustration of proteins using Phil Anderson’s original definition and showed that it is equivalent to the calculation of the number of interactions with null energy, using gaps in the alignment to gauge the zero of the interactions in the derivation of the energy from coevolution (Lui and Tiana 2013). Also frustrated contacts concentrate into few small, highly connected clusters.

The present data do not support the idea that the total number of frustrated interactions is minimised by evolution, as suggested by the principle of minimal frustration (Bryngelson and Wolynes 1987), but only that they tend to clusterise more. Of course, this does not exclude that minimisation of frustration could take place in the pre-biotic period.

## Supplementary Information

Below is the link to the electronic supplementary material.

Supplementary file1 (PDF 2403 KB)

## Data Availability

Upon request to the authors (large files).

## Code availability

https://github.com/guidotiana/Cocaine.
