# Supplementary material for: Evolution of frustrated and stabilising contacts in reconstructed ancient proteins
Source: Eur Biophys J. 2021 Feb 11;50(5):699–712. doi: 10.1007/s00249-021-01500-0 (PMC8260555; doi:10.1007/s00249-021-01500-0)
Supplement: Supplementary file 1 — Supplementary file1 (PDF 2403 KB) [file 249_2021_1500_MOESM1_ESM.pdf]

Supplementary Materials

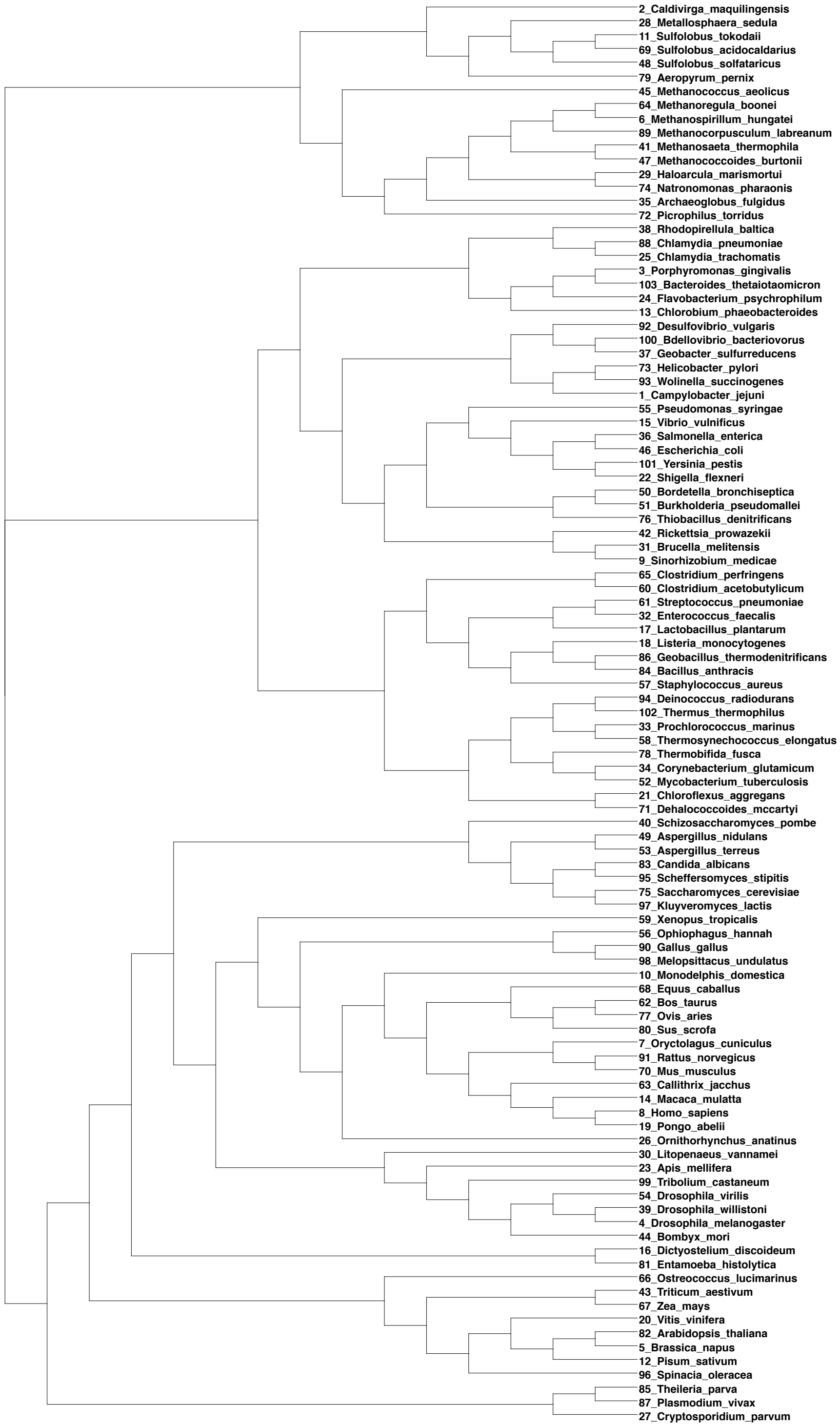

Fig. S1: Example of the phylogenetic tree of thioredoxin, obtained by TimeTree based on the organisms of the extant proteins, selected from Pfam (i.e., the leaves of the tree). Using PAML we reconstructed the sequences corresponding to the nodes of the tree (unlabeled in this plot). PAML is used setting the model F81(Felsenstein J, J. Mol. Evol. 17, 368, 1981) with an initial choice of the gamma distribution with α=0.4 and with ratio ω=0.4.

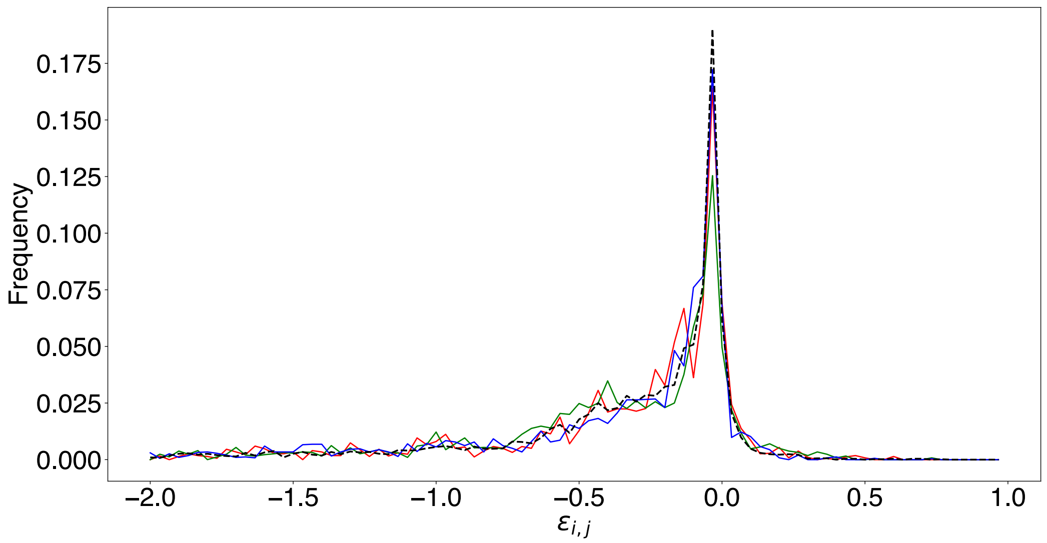

Fig. S2: Distribution of interaction energies of BLM averaged over all sequences of the family (black curve) and for three specific sequences (colored curves).

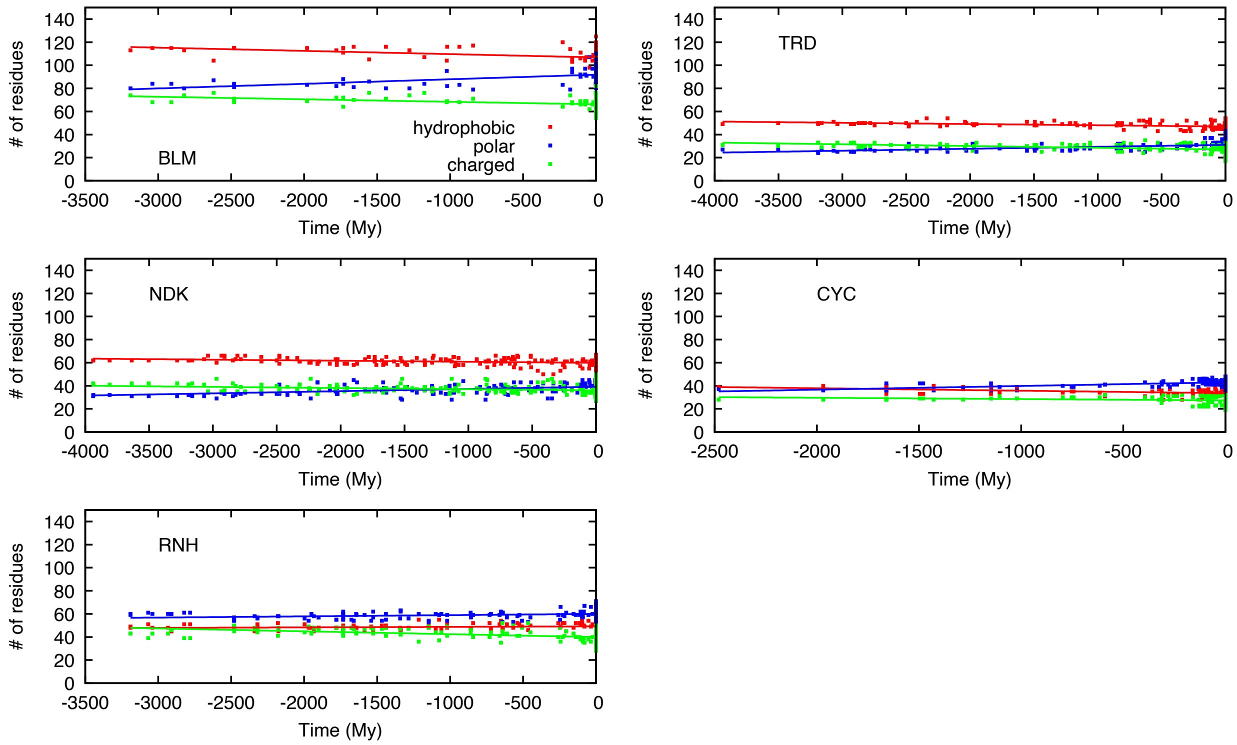

Figure S3: the number of hydrophobic (A,V,L,I,M,F,P,W), polar (C,S,T,Q,Y) and charged (D,E,K,R) in extant and reconstructed sequences. Solid lines are the linear fit of the points. None of the sets display a significant ($p<0.05$) temporal trend, as evaluated by bootstrapping.

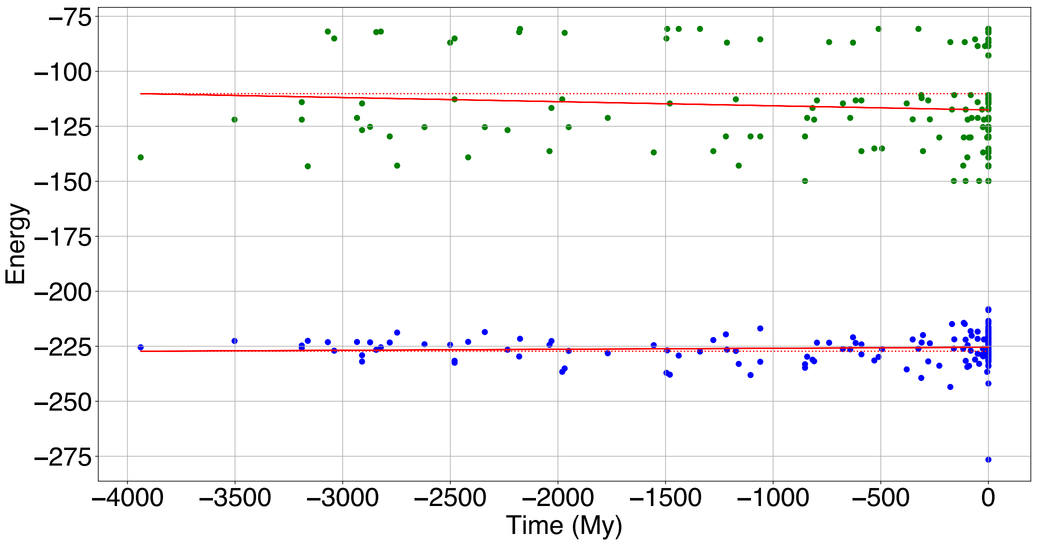

Figure S4: The native energies calculated for a random set of sequences with the same composition of the reconstructed ones for TRD. The red lines indicate a linear fit. The standard deviation on the slope of the fit, calculated from the random bootstrap is 2.5×10^{-5} My^{-1}.

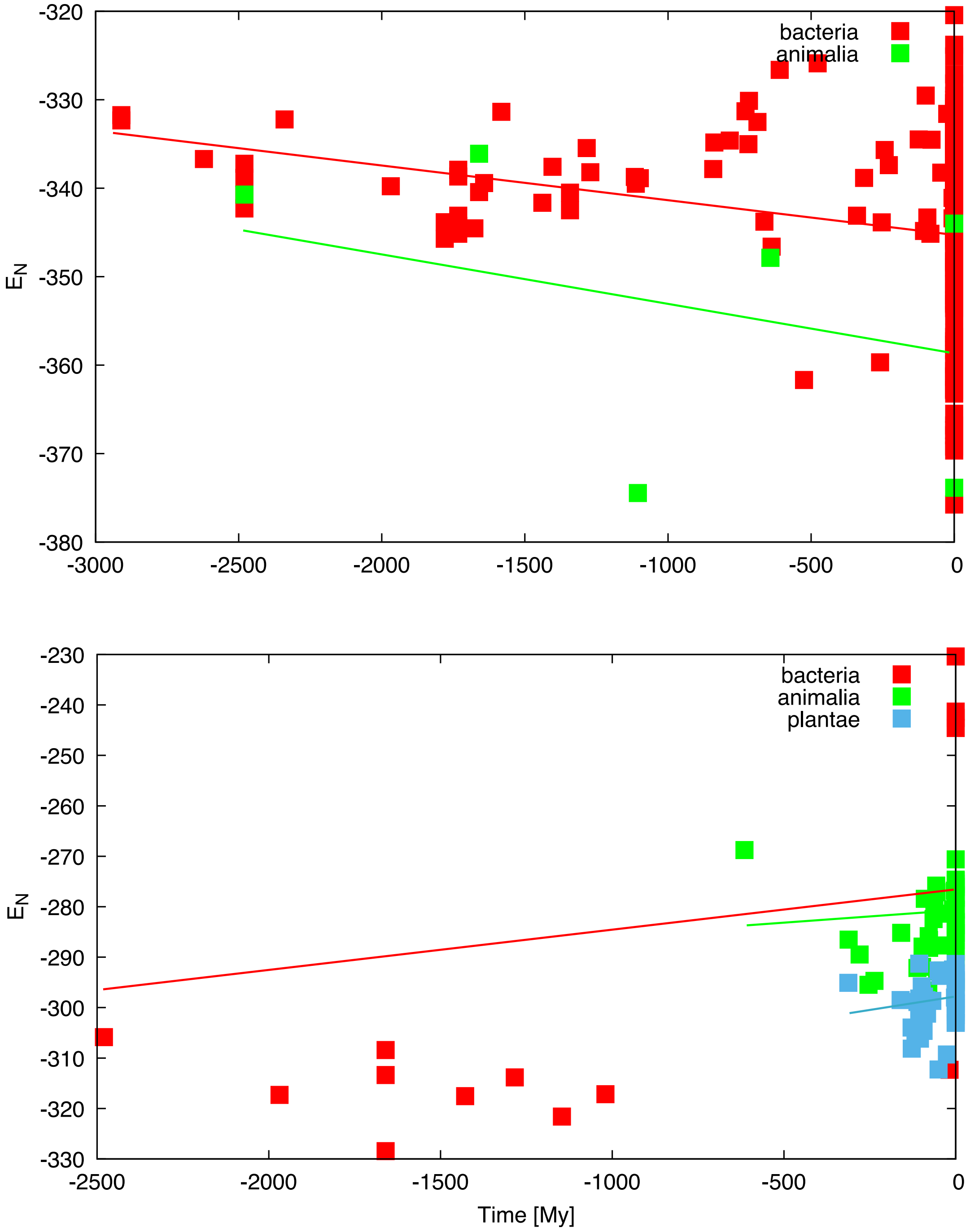

Figure S5: analysis of the native energy of proteins of the NDK (above) and CYC (below) family divided by kingdom. The elements of the different kingdoms display the same trend than the whole family. In case of NDK the average energy of animalia (-349.4) is slightly lower than that of bacteria (-338.4). In the case of CYC that of animalia (-281.7) is higher than that of bacteria (-318.1).

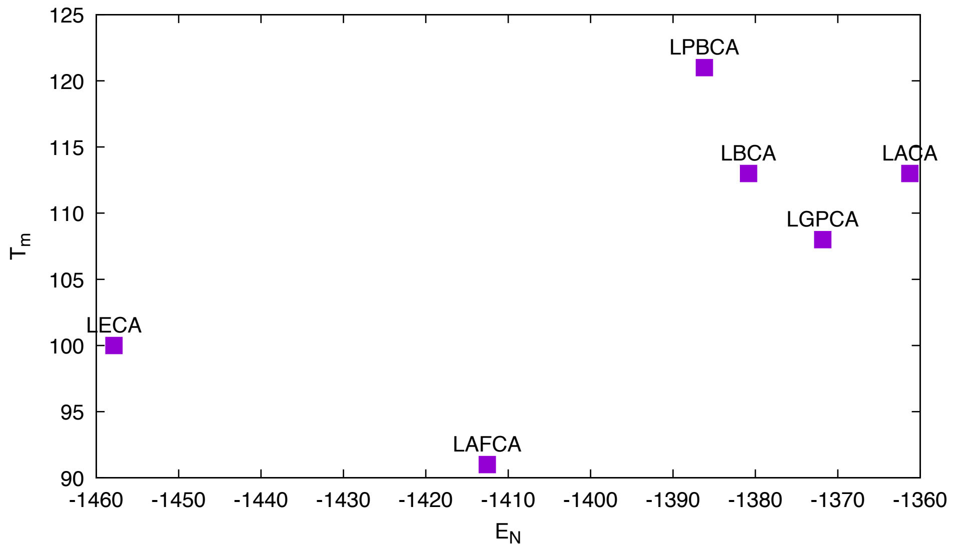

Figure S6: The experimental denaturation temperature plotted against the predicted native energy (cf. Fig. 2 in the main text) without filtering the interaction energies with the native contacts.

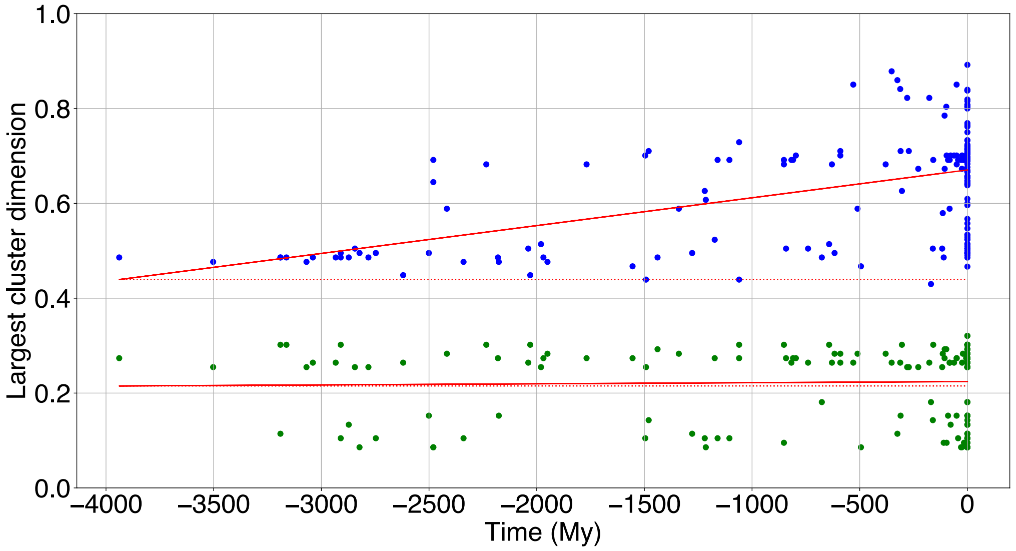

Figure S7: The size of the largest cluster calculated for TRD and for a random set of sequences with the same composition of the reconstructed ones for TRD. The red lines indicate a linear fit. The p-value of the data with respect to this null model is $<10^{-6}$.

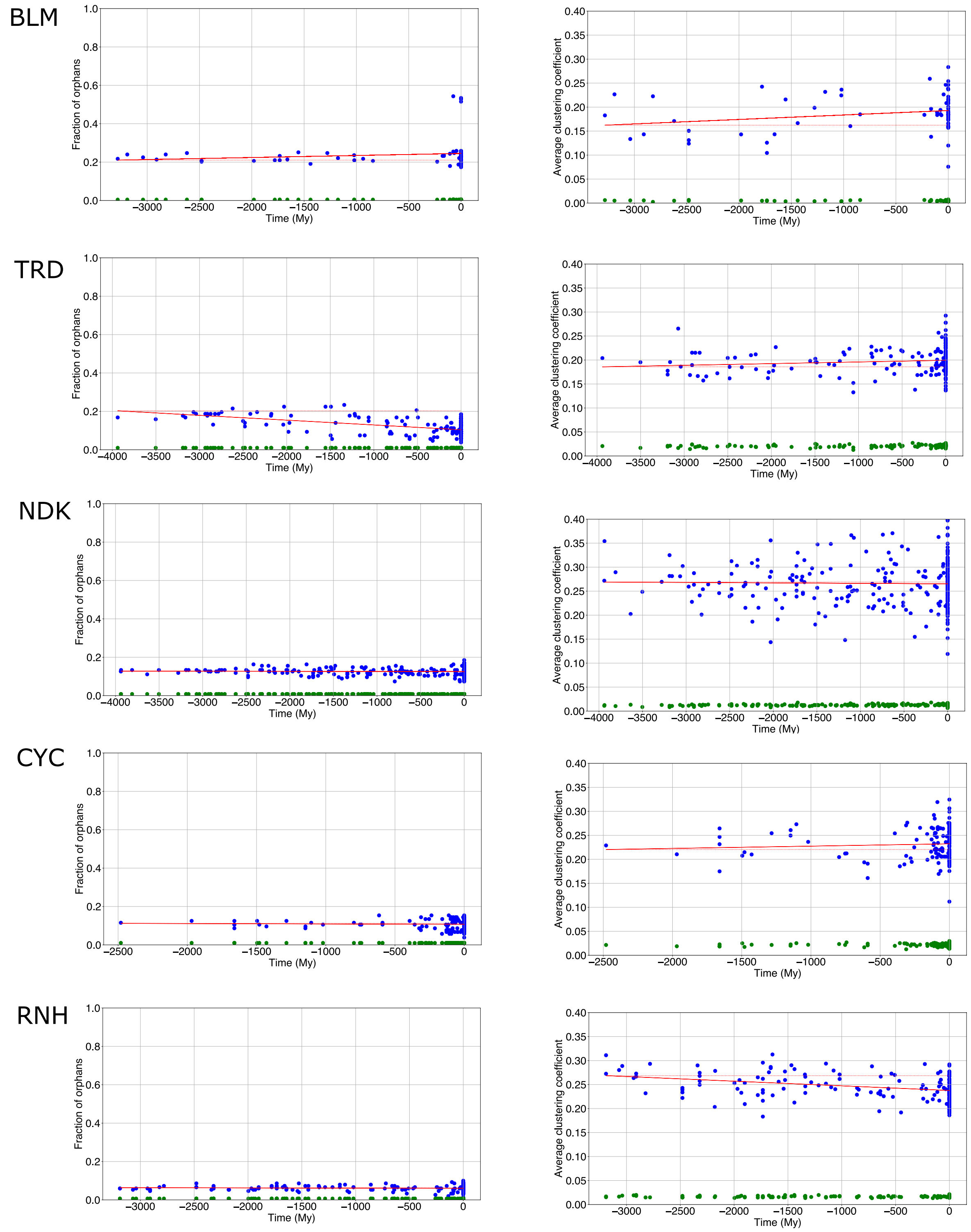

Figure S8: The fraction of orphans (left panels) and the average clustering coefficient (right panels) for the proteins belonging to the five families (blue points) and for the random bootstrap (green points).

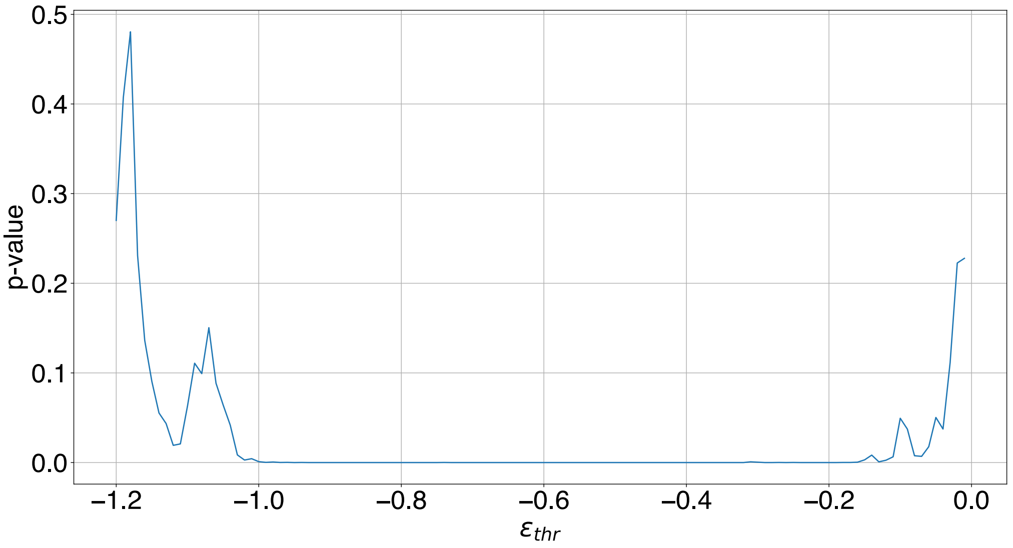

Figure S9: the p-value associated with the LCS of TRD calculated from a random bootstrap, as a function of the threshold used to define strongly attractive contacts.

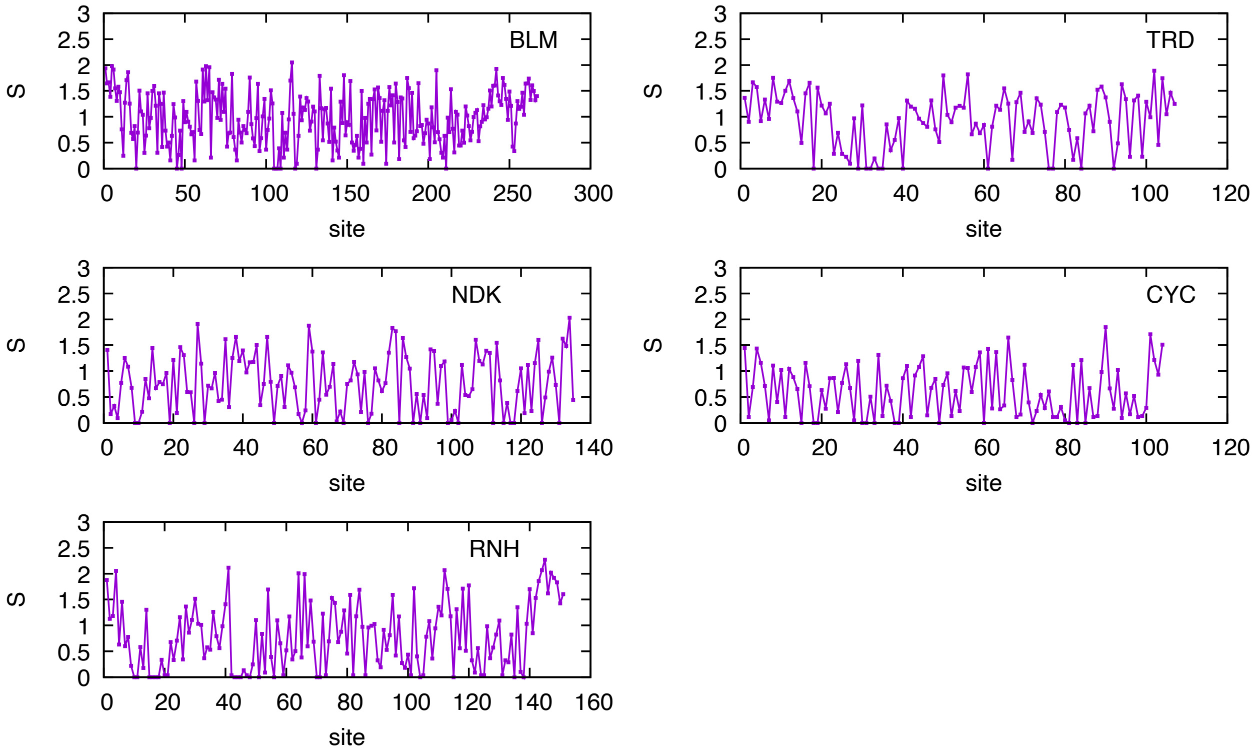

Figure S10: the mutational entropy per site, defined as $S_i = -\sum_{\sigma=1}^{20} p_i(\sigma) \log p_i(\sigma)$, being $p_i(\sigma)$ the probability that the sequence displays residue of kind σ at site i. The correlation coefficient of S_i with the mean interaction energy of Fig. 5 is <0.2 for all proteins.

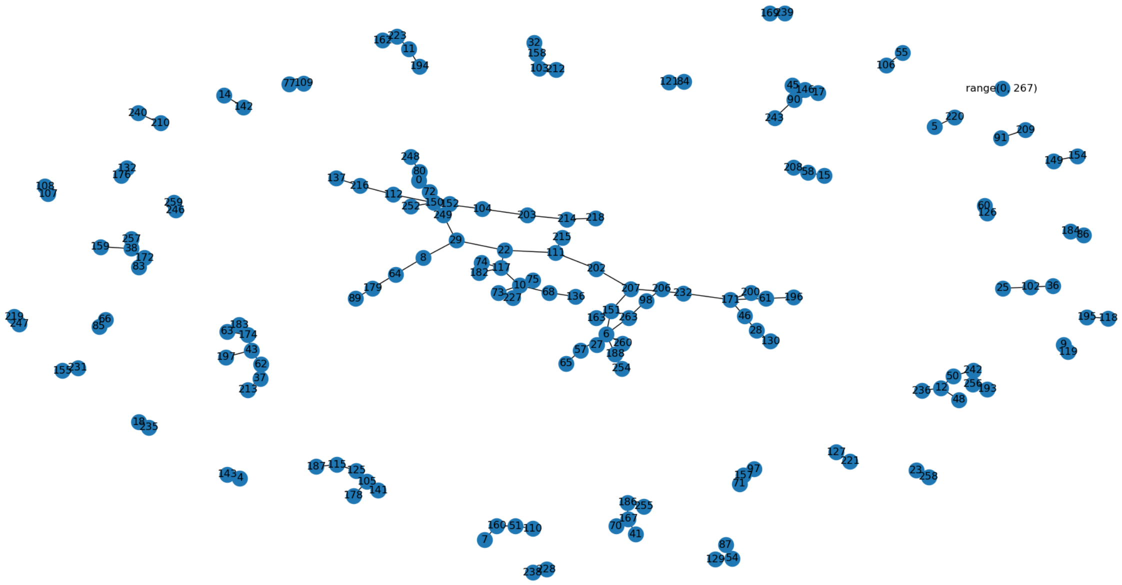

Figure S11: An example of random network of frustrated contacts, obtained by bootstrap of TRD.

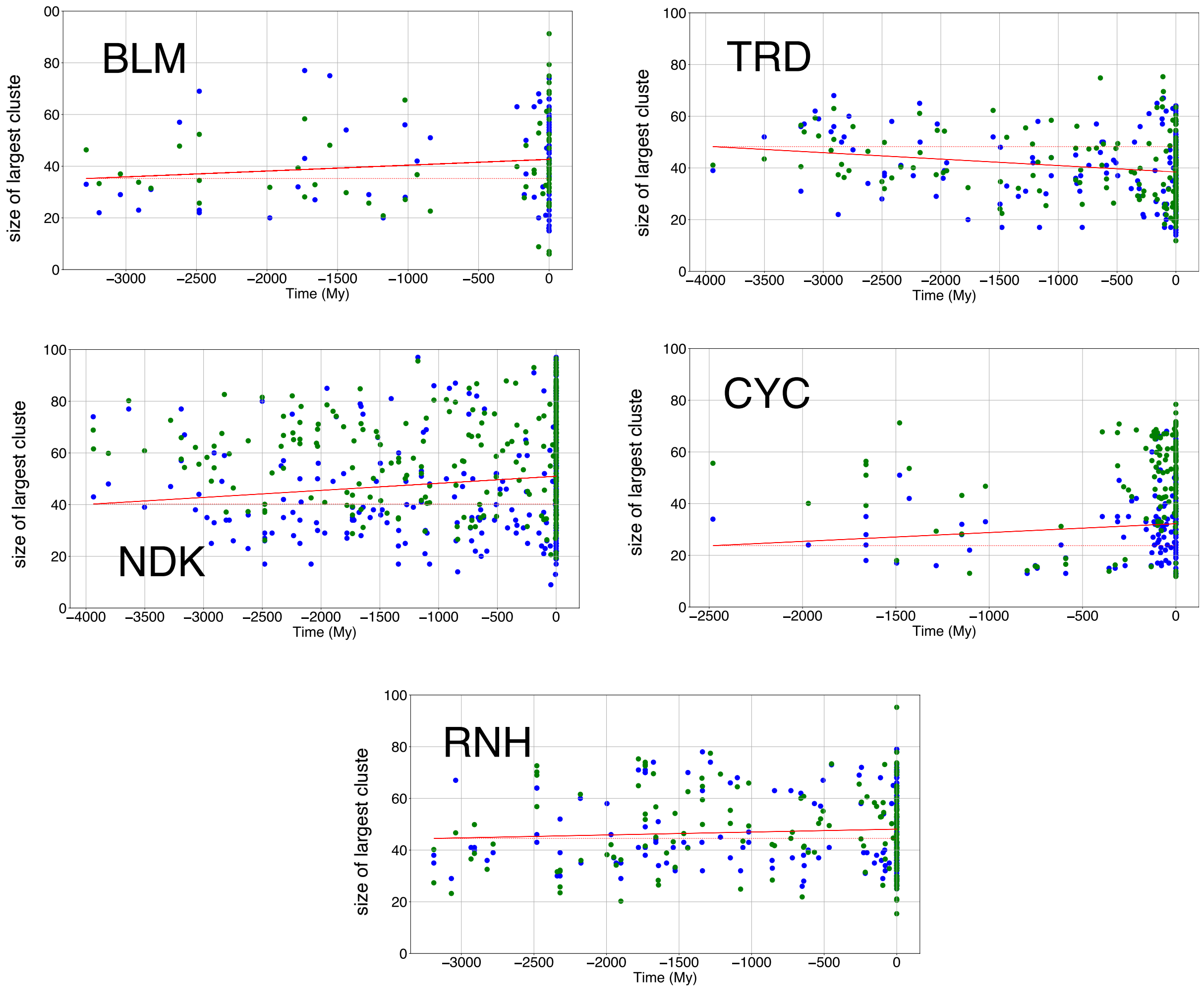

Figure S12: the size of the largest cluster of frustrated contacts (blue circles), compared with the average site of randomly bootstrapped frustrated contacts (green circles).

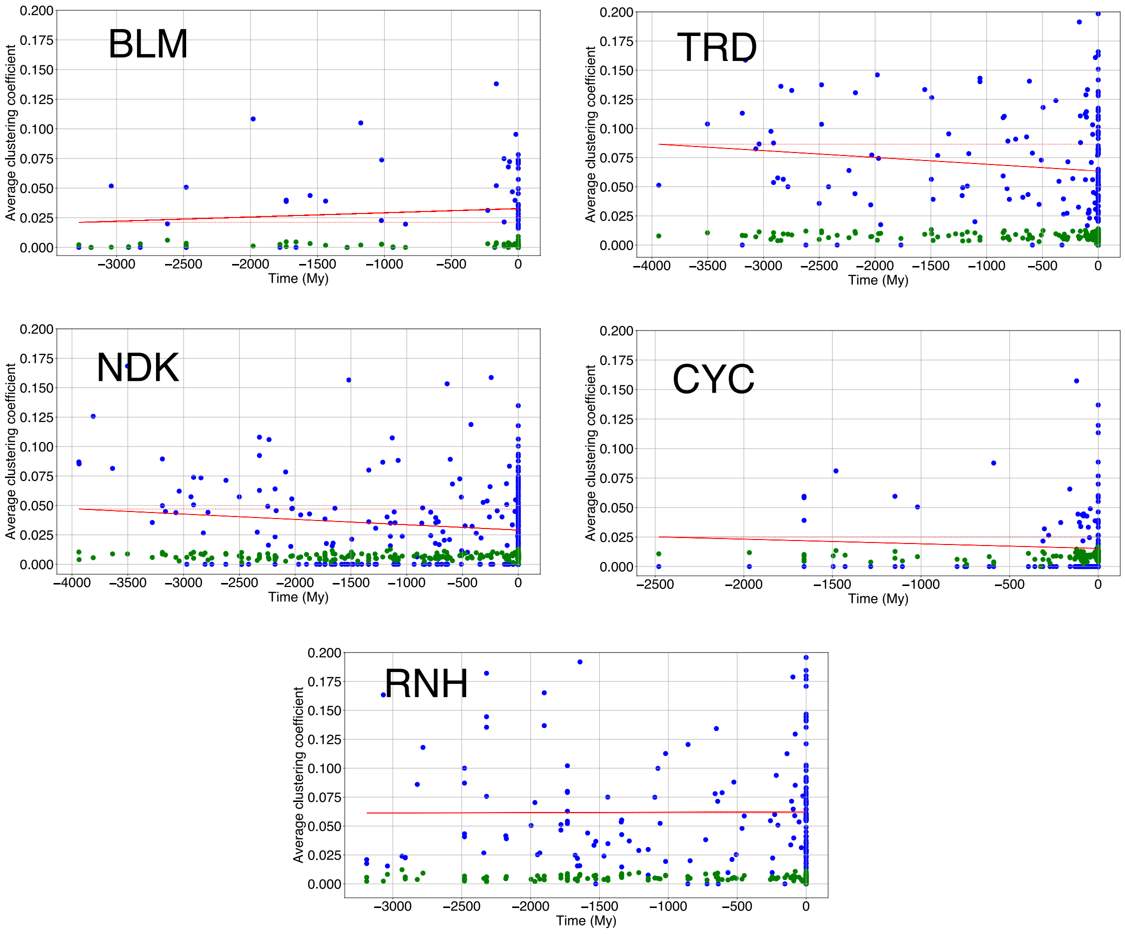

Figure S13: Clustering coefficient in the largest cluster (blue circles), compared with that of a random bootstrap (green circles).

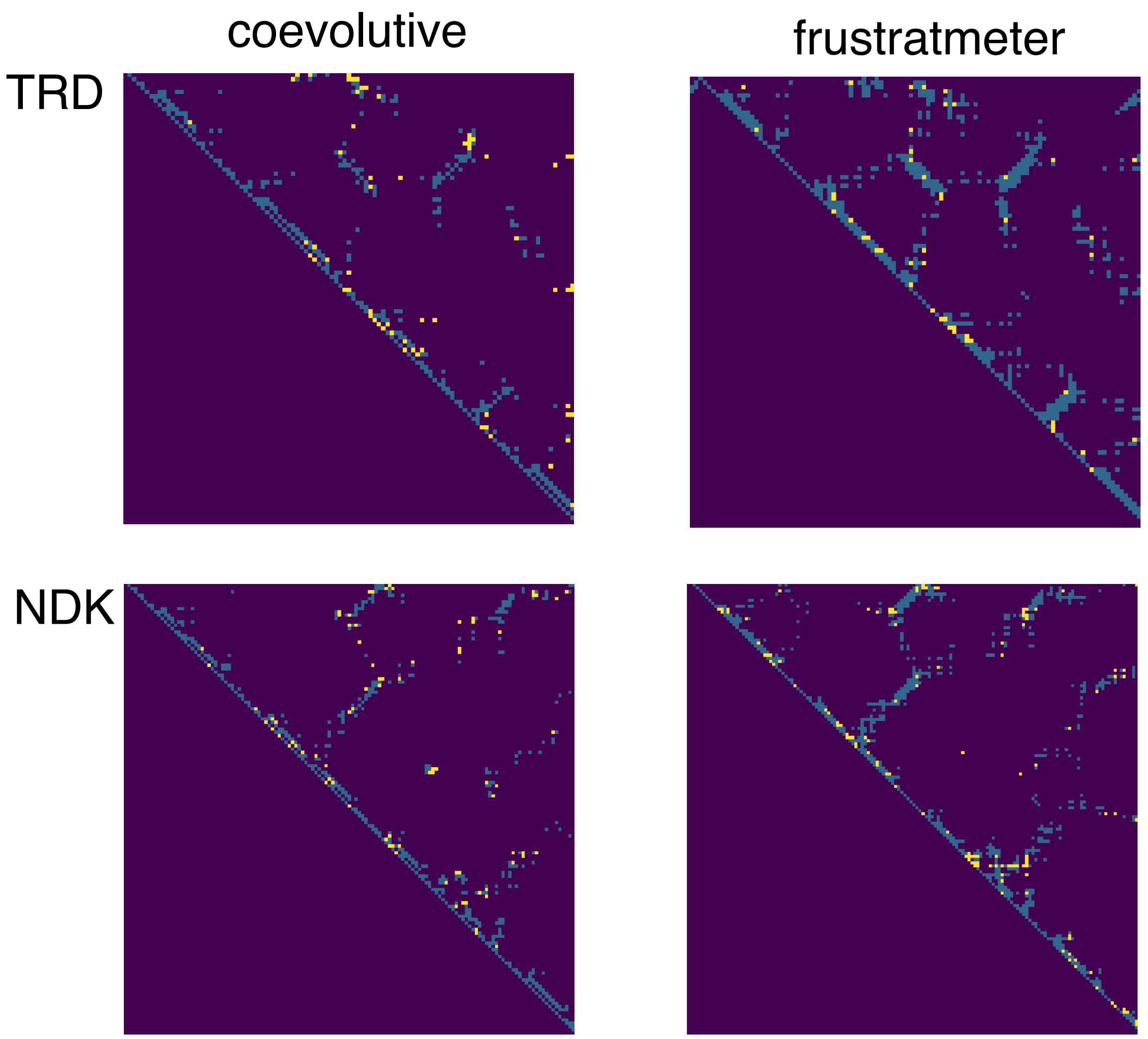

Figure S14: A comparison between the frustrated contacts (in yellow) calculated with the coevolutionary potential (left panels) and with the frustratometer ("sequence frustration", right panels) for a member of the TRD family (*Rickettsia Prowazekii*, upper panels) and NDK family (lower panels). Although they do not coincide pointwise, of the 64 frustrated contacts we calculated for TRD, 88% are within 3 residues of those calculated for the frustratometer. On the other hand, for *Bdellovibrio bacteriovorus* NDK (lower panels) the match decreases to 64%.

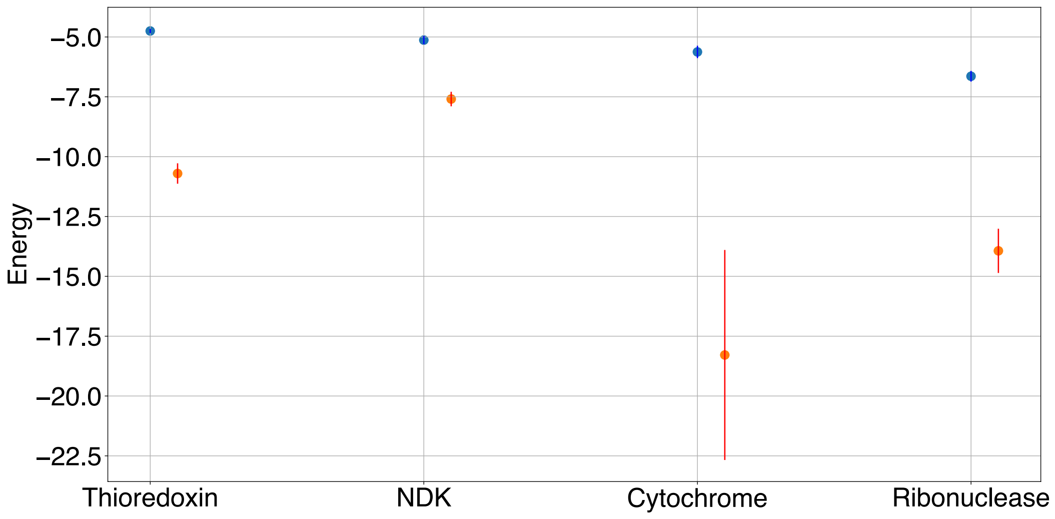

Figure S15: Average interaction energies of active sites (in red) as compared with those of all sites of each protein family. The location of active site is obtained from the Uniprot database.
